# Theories of disorder and order, energy and information, in sociological thought

**DOI:** 10.1098/rsta.2022.0292

**Published:** 2023-10-02

**Authors:** John Levi Martin

**Affiliations:** Department of Sociology, University of Chicago, 1126 East 59th Street, Chicago, IL 60637, USA

**Keywords:** thermodynamics, Gibbs, social theory, information, entropy

## Abstract

There has always been a close relation between thermodynamic theory and sociological theory, although they repeatedly part company and later rejoin. I discuss some of the most important ways in which the two have been in contact, focusing on the potential passage from theories of energy to theories of information and vice versa. I close by discussing how a closer engagement with classic thermodynamics may continue to be fruitful for sociological theorizing.

This article is part of the theme issue ‘Thermodynamics 2.0: Bridging the natural and social sciences (Part 2)’.

## 1. Energy and information, dispersion and organization

This will not be an intellectual history of the use of notions from thermodynamics in sociology; unfortunately, so far as I know, no such history yet exists, and I am not in a position to produce one. Instead, this is a selective discussion of ways in which the logic of thermodynamics has proven especially influential or relevant for sociological theory, focusing on the most prominent uses.

To begin, we have an unusual circumstance here in that the same basic mathematical formulae might be interpreted in very different ways. In particular, in social theory, there has been a distinction between those who use the template of *energy* as their paramount way of bringing thermodynamic equations or visions into social science, and those who use the template of *information*. (There is also a distinction between those in either camp and those who remained with a more mechanical view, but I return to that below.)

One might imagine that the concept of energy would be a straightforward one: for Aristotle, *energeia*, ‘being at work’, was contrasted with *dynamis*, the potential to be something or to do something. The actuality of energy thus led it initially to be less metaphysically freighted than this latter notion of *potential*—a reality that was not actual. But, when translated into early modern thought, *energy* became attractive to the more ideationally open, and hence more impressionistic, thinkers. This is because the mathematicization of energy had led to a recasting of the notion of *potential* as itself a form of energy: whatever it was, it certainly could be expressed in the same units as energy, even if one did not claim that it was literally a form of energy. Thus, absences (e.g. the absence of motion of a projectile at its apogee), so long as they are explainable in terms of an overall configuration, could have theoretical value.

This interest in potentials tended to distinguish those oriented to energy from somewhat more traditional (Hobbesian/Cartesian) thinkers who were more likely to use *momentum* as their archetype for thinking about things undergoing change. Although momentum is simply the derivative of kinetic energy, perhaps because of the absence of *potential*, those oriented to the Cartesian template frequently appeared to be more rigid and deterministic thinkers, while those oriented to energy tended to have a greater acceptance of poetic abstraction. The momentum-minded tended towards a billiard ball model of independent masses interacting via contact, while the energy-minded (e.g. [1, p. 241]) were more likely to think in terms of *configurations*, and it was this relational emphasis on configurations that became crucial for the generation of novel theoretical approaches.

In addition to these vague families of orientations, there was a second form of energy-based thinking in the social and behavioural sciences, one that focused on the putative zero-sum nature of energy. Conservation of energy was interpreted as a maxim for individuals and for groups: if psychic energy was dissipated, most worrisomely, into too much sexual activity (e.g. [2]), not enough might be left for undertaking bold civilizational plans, such as draining swamps and building bridges. Although this version of ‘energetics’ was influential in the nineteenth century, it lacked the capacity to provoke the elaboration of social theory, in part because adherents refrained from any real engagement with thermodynamics. Further, this vision of energy, often treating energy as if it were a mass of fluids stored up, as opposed to something generated by a particular configuration (akin to a potential difference), tended to be sociologically inert—leading to models of relatively self-contained units with limited battery charges, not the theory of the creation of voltage itself.

Interpretations of thermodynamic equations in terms of *information* came after those interpretations based on ideas of energy. Indeed, it was really only with Shannon's [3] important work on the theory of communication that information theory was born, and used to reinterpret some of the mathematical results from thermodynamics. Information, paradigmatically understood as a relation between two units across a channel, obviously was a theory ready-made for social thought, and the convenient assumptions that (i) talk was communication of information and (ii) communication of information could always be explicated as if it were a form of talk, led academics—who tend to talk a lot and may indeed think that the more talk, the more pay—to happily spin out theories in which communication or information was at the heart of social life.

Despite the obvious tendencies towards ideological distortion and tedious narcissism coming from the focus on communication (the replacement of *Homo faber* with *Homo blabber*), the information-theoretic approach turned out to be extremely generative. Like the energy approaches, at its core were fundamental results from statistical mechanics, results that also connected to the fundamentals of the statistics that were forming the method of the new emerging social sciences.

Yet there was a bizarre ambivalence about how to interpret these statistics, a rift in the first generation of sociologists that was barely understood, because interpretations of statistics were still being worked on. On the one hand, there was the interpretation that statistics was a theory of random error, and that error was equivalent to irrelevant and un-interesting departures from a fundamental social law. According to this line of thinking, statistics might help us correct our theory of the average, but everything, to the extent that it departed from the average, was a bit like Mary Douglas's [4] ‘dirt’—something not in its proper place. This interpretation was strongest in France and guided the development of orthodox academic sociology.

By contrast, there were others who saw the distribution itself as significant, while the average was unimportant, or even petty, compared with the extreme (that is, the positive extreme) of a distribution. This interpretation was stronger in England, where Francis Galton's adoption of statistics was used in furthering his quest to understand the nature of true genius, and in Germany, where philosophers often mocked the French for worshipping the average as the moral.

In other words, according to one way of thinking about things, all variation is basically error, and not social, and therefore not of interest for the development of social theory. According to the other way of thinking, the social is all about variation—the absence of variation is equivalent to the collapse of the social to a single point. Such a collapse—the end of the disagreeably unpredictable, disorderly and irrational action paths of their stubbornly independent countrymen—had long been seen as a valuable goal to French utopians. This tension over how to see variation was preserved in later work, including work in information theory, that honestly puzzled over the question of whether equiprobability was complete order, or complete disorder!^1^ Would a utopian world of complete equality be the ultimate achievement of the rational reconstruction of an orderly society, or equivalent to the heat death of a universe whose entropy had been maximized? Is all organization non-randomness, and is all non-randomness organization? These questions still drift around in social theory. To try to clarify, I will give a brief and selective recap of how thermodynamic ideas entered the social sciences, concentrating on sociology, and then consider what possibilities there are for future thought.

## 2. The early days of social statistics

It is true that early social scientific thought was inspired by physics, but it is not true that such thought was inspired by thermodynamics. In fact, the reverse is true: thermodynamics was inspired by social thought and took its ideas not from the mathematics whereby they had first been worked out in astronomy, but indirectly, via social science.

The famous law of errors, the inverse square law, had already been developed when the young Belgian scientist Adolphe Quetelet^2^ went to Paris to learn astronomy. He made the creative transfer of the mathematics he learned there, mathematics for the *repeated* observations of a *single* unit subject to error, to the different case of *single* measurements for *repeated* units. What is the difference, he said, between measuring a single statue 10 times with poor instruments and measuring 10 different people? In either case, we have a bell-shaped distribution with an average and a quantifiable standard deviation, and the interpretations can be similar. Nature, he said, shoots for the average man the way a marksman shoots for target—the variation that exists in our heights, chest lengths, chest widths, and so on, simply result from the fact that nature's gun has too short a barrel [9].

These ideas, especially as popularized by the Englishman Henry Buckle's *History of Civilization*, rippled through the educated world and proved to be vital for the development of statistical mechanics. Boltzmann and Maxwell both—apparently independently (in this paragraph I rely on [8]: 111ff)—used the analogy to individuals to explain their reasoning as they developed the statistical mechanics of gases. Boltzmann proposed that ‘The molecules are like so many individuals, having the most various states of motion, and the properties of gases only remain unaltered because the number of these molecules which on the average have a given state of motion is constant. The determination of averages is the task of the calculus of probability.’ Maxwell similarly pointed to the ways in which the social statisticians of his time were using census data. ‘The number of individuals is far too great to allow of their tracing the history of each separately, so that, in order to reduce their [statisticians'] labour within human limits, they concentrate their attention on…the varying number of individuals in each group, and not the varying state of each individual… But the smallest portion of matter which we can subject to experiment consists of millions of molecules, not one of which ever becomes individually sensible to us…so that we are obliged to abandon the strict historical method, and to adopt the statistical method of dealing with large groups of molecules.’

If techniques for social analysis could inspire thermodynamics, surely, there is no reason to be concerned if we let thermodynamics inspire social thought. Still, such cross-fertilization was not universally acclaimed. The two fields of inquiry only connected in the joint reliance on the probabilistic approach to aggregate statistics, and such ideas were not accepted by all social thinkers. First, we must remember that at the time, one could be interested in ‘statistics’—that is, information on populations that generally had been gathered by governments—and reject the notion that such human statistics were amenable to analysis via probability. Auguste Comte, the putative father of sociology, was probably not alone when he characterized this as a ‘radical inanity’ ([10, p. 492]).

Even more, despite the developing tie between these statistics and thermodynamics, there was (so far as I know) very little interest in thinking about the implications for social life of the thermodynamic laws of entropy. The assumption of progress as an obvious and incontestable feature of the globe, or of the west, or perhaps only of Anglo American societies, would likely have made any such inquiries seem silly. In fact, Herbert Spencer, in his *First Principles* [11, p. 327], emphasized the exact opposite law—what we might consider a law of reverse entropy. Take a beaker of a fluid warmed to a homogeneous temperature throughout and set it out on a table, Spencer directed. If you do, you will see that after a while the temperature is no longer homogeneous. This, he said, was an inherent law of nature—to go from indifferent homogeneity to organized heterogeneity. We might say that this was because Spencer was looking at one part of an open system, and he would have agreed. It is because one system interacts with another that they internally differentiate. Thus, the sort of systems theory being developed by Spencer—one based on the model of individual organisms struggling with their environment—at this point led away from any close engagement with thermodynamic thinking, and instead, towards a version of systems theory that would prove compatible with evolutionary biology (which only later was able to draw on the findings of statistical mechanics [for a review, see [12]]). This was to change in the first half of the twentieth century, at least among a small group of systems theorists.

## 3. Related influences from physical science

The Durkheimians, perhaps influenced by the work of the philosopher Émile Boutroux ([13, p. 158]; admittedly, Comte had had similar ideas), emphasized the distinctiveness of different sciences and their functional independence. No one science had to kowtow to any other, and no one would get points for importing principles from a more advanced science. This became the orthodoxy of the social sciences in the second half of the twentieth century, when the Durkheimian model, after having been stalled as a result of the wars, was really established and reimported into the United States and into England (there, it had already been institutionalized in anthropology; in the US, the neo-Durkheimian vision was actually put forward by Talcott Parsons, who claimed to be following the ideas of the very different Max Weber). According to this view, directly building on, or appealing to, or even simply importing ideas from a more advanced science, especially physics, was considered to be *très gauche*. However, there was always more methodological anarchy in theory building than there was in the definition of core methodological research practices, and there were various repeated attempts to ransack the sciences for good ideas. Given the centrality of statistical mechanics to other formal models in the physical sciences, I briefly note these directions, and how they opened potential avenues for the exploration of thermodynamic analogies.

Not surprisingly, some social scientists were enamoured of ideas having to do with relativity. They themselves had been earlier than the physicists in terms of climbing on the relativism bandwagon, and, even if one cannot completely accept Paul Foreman's [14] thesis that the interpretation of quantum mechanics as incomplete was due to general cultural trends in Weimar Germany, as opposed to following naturally from the mathematics, still, it certainly is true that general social interest in issues of relativism, on the one hand, and indeterminacy, on the other, fuelled the misplaced conviction with which some social scientists expected to be able to support some of their ideas by appeals to relativity theory, or to quantum electrodynamics, or both. Most such efforts were weak and stillborn, though there were some more mathematically sophisticated borrowings, including an early piece in the *American Journal of Sociology* on the use of Riemann surfaces for social thought! [15]^3^.

The interplay between the physics of energy and social thought for the case of economics—the partial affinity between theories of the conservation of energy (on the one hand) and economic principles of the zero-sum nature of exchange—has been told by Philip Mirowski [16]; here I wish to concentrate on a few moves towards accepting inspiration from the sciences related to thermodynamics that were to leave significant descendants in sociology. First, there were some who, like Mirowski's economists, thought that a social science could be built on energy flows. Although the anthropologist Leslie White [17] was hardly the first to say that human development involved the progressive harnessing of energy (think of Marx's theory of the continual development of the productive forces), he posited a direct relation between energy-*per capita* and cultural development, and insisted that social formations that could trigger large releases of energy with small (highly leveraged) inputs had an advantage over those that could not. More, his underlying vision of social life as sets of energy flows—a vision now returning to the new environmental sociologists and kindred spirits in sister disciplines—was inspirational to radical political economists looking for a way to nail down their belief in the inequality of economic exchange relations between the third and first worlds, a goal that had seemed to become unreachable with the abandonment of the labour theory of value (here see, e.g. [18]). This notion then entered into the sociology of development (e.g. [19]), where it joined other ideas about global flows of commodities, power, labour and, later, nitrogen, calories and carbon.

Second, there was an attempt to take seriously the lawfulness not of errors from a social law, but of distributions, most notably in the case of language by George Zipf, then generalized to other cases such as city sizes and incomes. This vision of distributions as inherently theoretically generative seems to have stemmed from statistical mechanics, although few seemed determined to work through the analogies. (Indeed, spatial and regional sciences, most importantly the work of Isard [20], suggested the tractability of certain systemic problems using a rigorous mathematical analysis, but the results were generally more complicated and substantively focused than most social scientists had patience for.)

One important case where ideas in physics that were thermodynamics-adjacent were being used to inspire social thought came in German Gestalt psychology (the best review is [21]). Although they were fundamentally influenced by the idea of *field*, and here directly drawing on Maxwell, Faraday and Einstein, their interpretation of the significance of the field for human visual perception first and foremost involved attention to configurations.^4^ The field that they were most interested in was what they considered a field of tension—*Spannungsfeld*—that had something akin to a distribution of potential energy. The optic system of an animal or human had a tendency to try to fall into a state of minimal tension. Seeing two near-semicircles almost touching was considered to have more tension than seeing a circle with an occluding but edgeless rectangle placed across them.

This notion of the field emerging via cascaded local interactions of domains not only built upon Faraday's interpretation of magnetic lines of force, but implied the possibility of a thermodynamic approach in several ways. First, if one were to treat these domains as units, and subject them to a law of the conservation of energy, one has a tractable thermodynamic problem. In other words, focusing on the nature of the potential energy in a field could bridge to some of the dynamics underlying generalizations of the Boltzmann model. Further, the very focus on local interactions suggested compatibility with a kinetic model. However, those attracted towards field theory tended to be those who were not enthusiastic about drawing on *mechanics*, which was associated with narrow-minded determinism. Even more significant for the development of this school, the very focus on the concrete and the intuitively accessible empirical research, and the eagerness of the émigré psychologists (most had to flee the Nazi regime) to respect the procedures and vocabulary of their hosts, led Gestalt psychology to mutate into a form of social psychology, divorced from the earlier neurological (and physical) speculations of Wolfgang Köhler [24].

The more audacious thinking inspired by analogies to science and thermodynamics came in the motley set of writers, and sometimes researchers, associated with systems theory (for example, [25]). This systems theory (the key work being [26]) drew from fundamentally biological understandings of systems, but wed them with a close reading of modern information theory. One, more technical, branch, influenced by the turn to examining the thermodynamics of open systems pioneered by Ilya Prigogine, was the ancestor of what became the complexity science in the Santa Fe model. Another, more impressionistic, entered sociology and anthropology as a mishmash of different ideas about systems and moving equilibria. It would be a disservice to posterity not to indicate that also included in this mix were general countercultural themes, number theory, interest in Eastern mysticism, the least tenable aspects of psychoanalysis and tens of thousands of micrograms of lysergic acid. What a surprise, then, that systems theory was at the same time being brought into social science by one of the more sedate characters in the postwar field, Talcott Parsons (first in [27]) at Harvard, claiming to be linking this to the mathematical visions of the great Italian economist and grouch Vilfredo Pareto.

Parsons—who had begun his first major work [28] by excoriating Spencer's vision of systems theory—later realized that he was only a stone's throw away from Spencer (see his introduction to [29]). Parsons's approach to systems theory turned on more or less generalizing economics in such a way that it had a greater variety of substances, and fewer equations. It was also loosely coupled with informational theory, although Parsons did occasionally appeal to ideas of energy and entropy and so on. It was his great disciple from across-the-pond, the German theorist Niklas Luhmann (e.g. [30]), who, deliberately drawing on one of those countercultural systems-type theorists, George Spencer Brown [31], attempted to bring information theory into sociology—and in a mathematically generative, if somewhat unspecific, way. This approach remains theoretically vibrant, especially in Germany, but has not yielded much advance in terms of formalization.

These various theoretical directions, then, represented some of the influential ways in which thermodynamic thought might be able to influence sociological theory.^5^ But at the same time that these theories were arising, and crumbling, and sometimes being revived, there was a more successful, and more direct, borrowing of thermodynamic principles in the social sciences. This involved turning to the mathematics of entropy to solve a number of different technical problems in social statistics.

## 4. Mathematical uses of entropy and thermodynamics

Thermodynamic thinking re-entered social statistics in two ways—descriptive statistics and techniques of model fitting. Regarding the former, entropy-based measures might be used to quantify *segregation* or its inverse, *diversity*. The long interest in residential segregation at different scales led some to use entropy-based measures, although the closely related Herfindahl was usually preferred for its interpretability. But entropic-based measures had an advantage in their theoretical clarity and their decomposability. First, they could be decomposed easily across hierarchically nested areas (see [33]). Second, they could also be decomposed into (i) the entropy at the level of the marginals and (ii) the mutual information between, which was theoretically relevant when the measures were used for subjective data (e.g. [34]). Ecology also made use of entropy-based measures for descriptive purposes, as these turned out to be good for quantifying the diversity in flora and fauna at different scales (e.g. [35]). A large family of related measures generalizing the Shannon entropy was developed and has also been applied to social statistics (recently, [36]).

However, there were also ways in which entropy could be used to gain insight on statistical distributions. The twentieth century saw the development of a coherent modelling framework for, first, categorical and then non-categorical, data based on information-theoretic perspectives [37], perspectives that could rival the Bayesian (e.g. [38]). The basic notion was that the mutual information of variables in a contingency table of cross-classifications could be used to reject certain hypotheses of independence. There was also a fascinating attempt to use pure combinatorics for at least certain data structures by Harrison White [39], but this, along with most of the information-theoretic approaches, was edged out by the computationally simpler version of loglinear analysis developed by Leo Goodman.

Still, there might be ways in which such information-theoretic approaches are, for some problems, more interpretable, or more theoretically generative, than loglinear models, despite the computational advantages of the latter (see, for example, [40]). However, the most important use of thermodynamic methods in statistics turned out not to be the ways of quantifying independence, but a much deeper engagement with core issues in the quest of solving complex statistical maximization problems.

The great problem in social statistics has long been the estimation of parameters of a generating model given a set of data. While it is child's play to construct the probability of any set of data given a stochastic model, going the other way—what R. A. Fisher called ‘inverse probabilities'—and finding the probability of a model or a vector of parameters given a set of data is quite complex.^6^ Thermodynamic principles turn out to be relevant for this work in two significant ways.

First, the problem of estimating parameters is often accomplished by trying to maximize the likelihood—to choose the parameters that, of all such parameters in a set, maximize the likelihood that we would observe the data that we *did* observe. This can also be understood as minimizing the deviations of the observed data from those that would be predicted under the model (whether through minimization of a penalty function like an *R*^2^ or some other criterion like the likelihood-based deviation, or indirectly via maximization of the likelihood itself). But this turns out to be very similar to the project of maximizing the conditional entropy of the ensemble subject to certain constraints. (And in one particular class of relevant distributions the maximum entropy *is* the maximum likelihood.) Further, in information-theoretic terms, we can also quantify the degree of difference of a set of data from theoretical predictions in terms of the information divergence ala Kullback.

Many of the methods that were developed to estimate such parameters (and their standard errors) worked quite well on sets of data in which the units could be assumed to be statistically independent, but were not necessarily unbiased in more complex data structures. Such data structures commonly arose in spatial settings, but also in non-spatial networks, in which some units were interdependent in some ways upon (at least) their neighbours. Here, it proved possible to rely on fundamental thermodynamic models to find alternative ways of making such estimates.

Specifically, let us return to the Boltzmann equation (or the ‘canonical ensemble’), which, as we recall, was inspired by Quetelian social statistics. This links the probability of the *i*^th^ state to [1/*Z*]exp(−ε*_i_*/*kT*), where *ε_i_* is the ‘energy’ of the state, *T* the temperature, *k* Boltzmann's constant and *Z* is a normalizing constant, the sum of the main term across all possible states. However, the intractability of coming up with a closed-form solution for *Z* in most cases means that the actual probability of any state cannot be calculated. Even could this problem be solved, attempts to use random simulation (Monte Carlo methods) of possible states become implausible for high-dimensional cases, as almost all of the probability mass is concentrated in one area, and thus almost all randomly chosen points have negligible probability, and do not contribute to a solution. Somehow, there has to be a more guided exploration of the space.

Metropolis *et al*. [41]^7^ realized that it was possible to produce the probability distribution over all *i*, and hence any of the descriptive statistics for such an ensemble (which are weighted by the probability and appear as an integration problem), by setting up Markov chains that would go from one state to another according to a symmetric function based on their *relative* probabilities (no need to estimate *Z*). If the new state *j* was of lower energy, the system would shift to that state, but if the new state was of higher energy, it would shift with probability exp(−[*ε_j_* – *ε_i_*]/*kT*). They demonstrated that the system's probability distribution would asymptotically approach the Boltzmann equation.

Boltzmann had turned to combinatorics to develop this fundamental thermodynamic result precisely because he understood that there was a non-independence across the various particles in an ensemble of a given energy. And this basic approach turned out to be vital for dealing with tractable cases of statistical non-independence. The key development that brought this into social statistics (though quite slowly) was the Hammersley–Clifford theorem (here, like everyone else, I rely on the ‘simpler’ proof worked through by [42]). By definition, it is possible to factor the ratio of two different joint probabilities as a ratio of the products of each's conditional probabilities. This is then also true if the distribution in the denominator is the probability of the null state (all observations zero); this ratio then becomes the key quantity at hand.

Now, by the Möbius inversion lemma, this ratio can be factored into a large set of terms pertaining to all the different possible subsets of the observations—leading to a sprawling set of terms too large to be tractable. However, if the observations are arranged in such an order that some are neighbours of others, and that non-neighbours are conditionally independent, it can be shown that all the subsets that include at least one pair of non-neighbours cancel out, leading to a much simpler factorization. This then can be shown to imply an exponential distribution with terms for the cliques of non-independent observations, which turns out to be equivalent to the Gibbs notation for the Boltzmann equation. Because of the restriction of the non-independencies of the overall configuration to those originating from neighbour relations, the ensemble is known as a Markov Random Field, and the Hammersley–Clifford theorem is thus glossed as ‘if some *X* is a Markov Random Field, its probability function may be written as a Gibbs distribution, and vice-versa.’

This led to the development of Markov Chain Monte Carlo techniques for estimating posterior probabilities, and the explosion of applied Bayesian methods (some of this story is told in an unpublished MA thesis by Tiani Li [MAPSS, University of Chicago, 2022]); however, these techniques were generally used in the social sciences to better estimate existing classes of models whose theoretical bases were independent of the underlying physical model involved. The one possible case in which there might be theoretical cross-fertilization would seem to be the field of social network analysis. This is because the same mathematics that underlay the processes used to *fit* the model also underlay the model itself.

Frank & Strauss [43] realized that the Hammersley–Clifford theorem also had implications for the attempt to construct probability distributions for social network data, a project that had engaged many of the most creative minds in social statistics (here, see, e.g. [44]), but had run into problems identical to those bedevilling Metropolis *et al.*, namely the determination of the *Z* constant allowing for the estimate of absolute probability. Making assumptions as to the homogeneity of indistinguishable structural states led to what was first called the p* model, a family of probability models for social networks marked by the Markov assumption (e.g. [45]). The estimation first employed—pseudolikelihood, also developed by Besag [46] and adapted by Strauss & Ikeda [47]—turned out to be have greater bias than expected. The natural response was to attempt to follow Metropolis and others and estimate the *Z* (or get around requiring such an estimate) via brute force, by using the ratio of probabilities to generate Markov walks not on the original Markov field, but on the network of possible states.

But why would this make any sense? The Metropolis algorithm was developed to estimate integration problems over ensembles of particles, e.g. pressure of some gas at a certain volume and temperature, not to estimate parameters of a model. But Geman & Geman [48] made use of the Hammersley–Clifford theorem to solve a practical problem in image restoration—given an image realized with noise, how do we recreate the true underlying scene of which it is an image? This can be understood as a question of inverse probabilities on a Markov Random Field, if we assume that any pixel is independent of any other conditional on its relations with its immediate neighbours. Rather than maximize the likelihood of the observed data given the model, they took a Bayesian approach and attempted to find the mode of the conditional posterior probability of the underlying ‘model’ conditional on the data. Since, for any degree of average energy, the Gibbs distribution can be derived by maximizing entropy, this turned out to be a convenient way of solving maximum entropy problems [48, p. 727].

This was a breakthrough in generalizing the application of these thermodynamic models more widely, but it may also contain a useful template for visualizing future theories, as we tend to be most comfortable using visual metaphors and cases of planar surfaces for organizing our thoughts about social life. I am thus going to close by suggesting that social science may find theoretical inspiration in these methods—the Gibbs distribution and its underlying composition—just as Quetelet found inspiration in the Gaussian curve and its underlying dynamics.

## 5. Theory now for the future

In a way, the curses of the current social sciences are twain. First, there has been the capacity to envisage all problems as variations on a form of equation in which a set of estimable parameters and observed data on the right-hand side generate predictions on the left that can, in turn, be compared with data. The realization that frequency tables with a multinomial distribution could be recast as models for frequencies following a Poisson distribution killed off one of the last remaining alternative ways of thinking, one that inherently tended to lead to a focus on the joint distribution of the given data as opposed to the (hypothesized) generator of individual observations. Now almost all statistical methods follow Quetelet and privilege as the theoretically crucial entity the (conditional) mean—the variation *around* the prediction is simply an error distribution often chosen for reasons of mathematical convenience or empirical fit.

The second curse has been computing power. It has become nearly trivial to estimate more or less any mildly identified model that one can write down, and hence there has been an explosion of terms on the right-hand side. The result is not only thousands of incomparable ‘models’ that really are bereft of any strong interpretation, but a mistaken vision of science as progressing by increasing the number of concrete determinants—as if Galileo could be criticized for only thinking about point masses, and not also including balloons, feathers and magnets when he did his (fictional) experiments [49]. This has led us to prefer models that seem to possess realism, but interfere with our capacity to understand more elegant, and more theoretically generative, simplifications that might speak to the nature of distributions.

Consider the theoretical clarification that came after Quetelet's development of the statistics for social phenomena. He was struck by the fact that a predictable mathematical shape arose in a population. He had made a reasonable stab at explaining the compatibility of this putative lawfulness and free will, but his ideas could not but give rise to the notion, put forward by his popularizer Henry Buckle, that, e.g. a certain number of people *had* to kill themselves, and if one resisted the dark urges, another would find them all the more pressing. Even Emile Durkheim was not free from this interpretation.

But Wilhelm Lexis pointed out that this was a fundamental misinterpretation of the nature of the distribution. If there was actually a non-independence such that the individuals could not be seen as completely free, it would appear in the form of under-dispersion (a tighter distribution than generated under the simplest Gaussian process). The very simplicity and elegance of most of the curves Quetelet studied should reassure us: ‘No one is therefore, for example, required to hang himself, in order to complete the budget for suicides’ ([50, p. 19]).

And, indeed, in most sociological distributions, we find *over*-dispersion, indicating that, relative to our paltry understandings, what we are missing is less external shaping, and more the heterogeneity of our samples. Such a result seems to me far more theoretically generative than knowing to what extent the income of college graduates born in the Netherlands to the same parents but differing by one year of education will vary.

Could we continue to gain theoretical insight not from predicting particular cases, but focusing on the theoretically generative aspects of different distributions? Although there has long been interest among statisticians in using over-dispersion as an indication of the influence of omitted variables (though this can also be true for under-dispersion), such tests are usually conducted for purposes of robustness—not as a way of pursuing competing theoretical visions. One case, however, in which this has been done pertains to the ‘log-log’ (or ‘scale-free’) distribution, which arises under conditions of what Merton [51] called the ‘Matthew effect’—those where current increase is a function of past stocks: large cities grow faster than small, people add links to popular websites proportionally more than they do to less popular sites, and so on.

It is true that one can indeed have a scale-free distribution where such dynamics are *not* present (indeed, as the name ‘scale-free’ indicates, it can arise simply because the data are collected from units of widely different scales); eyeballing distributions is rarely an important technique.^8^ What is more important is theorizing the antecedents, processes and consequences that are associated with the arising of certain distributions. For many of the phenomena most of interest to social scientists—political polarization, inequality, forms of mismatch—are inherently distributional issues.

Take the case of income inequality, often examined in sociology using thermodynamic-based entropy measures of concentration, but with no clear theoretical argument. Imagine that we were to actually propose that the distribution of incomes was akin to a Boltzmann distribution (for example, see [53]), and that economic upturns—perhaps especially those coming from directions that could be considered exogenous to the economic system (the classic though controversial example in European political economy was the discovery of the silver mines in South America as part of the colonial invasion). We might propose that the distribution of incomes *p_i_* would shift in accordance with a raising of *T*, yet tend to assume a Gibbs distribution. If we went further, we might propose that the significant characteristics of this distribution would be wholly due to the non-independence of certain configurations.

In no way am I suggesting that we should be inattentive to microinteractions. But often we move theory forwards more when we come up with stylized interactants from equivalence classes defined by well-understood properties, and not when we attempt to do justice to the full empirical richness of cases. It was attention to the underlying kinetic relations of gaseous molecules that allowed Maxwell not merely to derive distributions of the velocities of particles and of the free paths travelled, but to come up with the very counterintuitive conclusion that the friction within the gas should be independent of density—something Maxwell himself initially imagined could not possibly be true ([8, p. 117]).

Of course, as they say, Garbage In, Garbage Out—theories that begin from *false* axioms are bad theories, but there is a difference between *partiality* and *falsity*. Rational action theory has proven a good theory for cases where its partiality is conformable to the institutional strictures that shape action, and less than useless where it does not. We might also want good theories that ignore certain aspects of reality so as to proceed unencumbered by complications when these theories then allow us to develop non-trivial and testable conclusions—those that are at variance with the conclusions drawn from *other* sets of plausible axioms. I would like to end by sketching, hastily and roughly, one possible way in which we may be able to draw renewed inspiration from thermodynamic theory for sociological thought.

One of the difficulties with using ideas from statistical mechanics is that they appear in so many different guises that it may encourage an undisciplined assimilation of different substantive domains to one another, leading to a mushy porridge. Still, with suitable caution, it might be worth taking seriously the interchangeability of *energy* and *information* for some purposes.^9^ Here, the exciting implication would not be that the second law of thermodynamics implies that we are moving to a world without meaning or knowledge. Although (as Leo Szilard proved in 1929 [55, p. 139]) it is true that the energy loss required to gather information can be theoretically significant (it provides the solution to the puzzle of Maxwell's demon), any facile critique of systems theory as an interchange of information that must actually end in heat death (or brain death) takes us in the wrong direction: no one denies that the earth is an open system, and hence may have increasing local organization. Instead, the interesting direction might involve a somewhat awkward bridging of thermodynamics and field theory, for if a field is indeed a set of organized potential energy, it may also be understood as a latent capacity to generate information. (Compare the different be related, and inspiring, ideas of [56, p. 213].)

A field can organize a distribution that, in aggregate, may also be compatible with a Boltzmann–Gibbs model. Of course, a field is not actual energy, but potential energy. But rather than positing merely a single macro distribution, a field involves structured variation in the strength of the potential. What might be a possible way forwards is to consider some ways in which the same overall distribution, and the same total field strength, can be differentially organized. Perhaps the simplest such approach is the range of order (to what extent nearby areas' potential vectors are similarly oriented), which could be (after temperature) a second macro property that is theoretically generative (here see [57]).

But it might be that we can go further in taking seriously the notion that a field is an organization of potential energy. Randall Collins [58] has proposed a theory of the generation of ‘emotional energy’ as a general template for thinking about social action; this theory has, at the most general level, certain homologies with the field theory of Pierre Bourdieu, which also is in some ways a generalization of certain action theories (for those unfamiliar with his work, the best starting place is [59]; the technically field-theoretic nature of Bourdieu's approach is defended in [60]). Collins envisions actors going from one ‘interaction ritual’ to another, with successful interaction rituals increasing the emotional energy of at least some participants. The phenomenological, indeed, physiognomic, plausibility of an account of emotional energy is impressive—we not only feel buoyed up in some situations, but depression and stress take their toll on the physical structure of the body.

However, if we take the notion of emotional energy as literally as we can, we must puzzle over the question of how it is *stored*—the person who was buoyed up returns to calm, but may re-start social interactions the next day with more confidence.^10^ Perhaps this question turns out to be the same that a rigorous field-theoretic interpretation of Bourdieu would begin with: How does the potential energy in fields develop? While recognizing the great difference between the uses of ‘field’ in these two senses (that of classic field theory and that of a Markov Random Field), it might help to imagine the sorts of interaction rituals that Collins discusses as specific social groups that are technically cliques, and that the total social energy of some social order can be partitioned into that associated with such different cliques in a Gibbs distribution that is correlative to a Markov Random Field. Such an outrageously abstract claim might seem worse than useless, if it only gives us the sense that we have precision where in fact we are completely at sea. But it might also suggest the key issue for social research: to find something that in analogous to the ‘displacement currents' that Maxwell deduced—ways that a slightly compressible fluid might hold energy, even if it involved electric currents existing in empty space. It might be that the equivalence between energy and information could allow us to, vaguely and impressionistically at first, hopefully more precisely with time, understand how human action shapes these sorts of configurations.

## Data Availability

This article has no additional data.

## Footnotes

1 A similar confusion also arises for some in the fact that the classes of indistinct states that are the ‘most probable’ (in that they are most likely to be observed) are not necessarily those with the highest ‘probability’ (greatest entropy), since there may be more equivalent configurations that lead to somewhat lower entropy states than the single possible macrostate of maximum entropy. It is unlikely that we would get something *that* likely.

2 Excellent sources for the history of statistics and the relation across sciences are Stigler [5], Hacking [6], and the various essays collected in Kruger, Daston & Heidelberger [7]. However, my chief source for the relation between thermodynamics and social statistics is Porter [8].

3 As far as I can tell, Hewes was a student of Albion Small's and made this amazing argument in class that impressed him so much that he asked her to write it up for the journal—he was, I believe, desperate for material.

4 While economists—most importantly ([22]: 5, 9, 17f)—understood the relation between their use of energetic/hydrodynamic metaphors and a field analysis, Mirowski's [16] point is that the weakness of their approach resulted from not more rigorously following through on this insight, and deriving conservation from the nature of a conservative field. Some of the Gestalt theorists, especially Köhler [23], did.

5 There were other uses of thermodynamics in sociology, some in Eastern Europe, and one in the West Coast of the US, as Kenneth Bailey tried to go further than Parsons in developing a general systems theory that had some relation to entropy (for a brief statement, see [32]), but, like Parsons's, lacking a real mathematical connection to actual data, the effort did not influence others.

6 Note that not all statisticians or epistemologists agree that a model *can* have a probability; assent to this plank is usually restricted to orthodox Bayesians and those who have not really thought about it.

7 The Metropolis algorithm got its wonderful name by the fact that Nicholas Metropolis controlled the computer at Los Alamos, but more central in the development of the method was the last author, Edward Teller, father of the H-bomb and inspiration for the insane physicist in the movie *Dr. Strangelove*. The computer they worked on was named MANIAC. As Fred Kwan said, ‘It's the simple things in life you treasure.’

8 Another case in which distributions were seen as more theoretically generative than they actually were involves the logistic growth curve and scientific developments. For a discussion of these issues, see Martin [52].

9 There are a few theorists who have understood the theoretically generative nature of the fact that one may move between considering non-independence as a flux of information or a flux of energy, and that we are free to layer an informational interpretation on top of an energetic distribution. Here see Granger [54].

10 It was Benjamin D. Zablocki who brought this issue to my attention.
